# Complementary and Alternative Medicine (CAM) Use among Elderly Cancer Patients: A cross-sectional study in South Korea

**DOI:** 10.21203/rs.3.rs-7605426/v1

**Published:** 2025-09-19

**Authors:** Hyunyem Chang, Soo Jeung Choi, Hyea Bin Im, Dain Choi, Dongwoon Han

**Affiliations:** Veterans Hospital System medical center; Hanyang University; Hanyang University; Hanyang University; Hanyang University

**Keywords:** Complementary Therapies, Neoplasms, Aged, Republic of Korea

## Abstract

**Purpose:**

Complementary and alternative medicine (CAM) use is common among elderly cancer patients, but evidence on its prevalence, characteristics, and determinants in Korea is limited. This study aimed to examined prevalence, patterns, reasons, and associated factors of CAM use among elderly cancer survivors.

**Materials and Methods:**

A cross-sectional survey was conducted with 420 cancer patients aged ≥ 65 years attending outpatient clinics of the Veterans Hospital System, a 1,000-bed secondary hospital in Seoul, Korea. Data on CAM utilization, modalities, reasons for use, and information sources were collected through structured questionnaires. Multivariate logistic regression was used to identify demographic and clinical factors associated with CAM use.

**Results:**

Among participants, 60.0% reported CAM use. The most common modalities were exercise (fast walking, 49.4%) and dietary interventions (42.9%). The primary reason was immune enhancement (61.6%), and family members or relatives were the main information source (42.1%). Multivariate analysis revealed that being married (OR 2.49, 95% CI 1.40–4.45), having prostate cancer (OR 2.14, 95% CI 1.36–3.35), and undergoing surgery (OR 1.62, 95% CI 1.07–2.45) were significantly associated with CAM use..

**Conclusion:**

CAM use is highly prevalent among elderly Korean cancer patients, particularly in married men, prostate cancer patients, and those who have undergone surgery. Oncologists should incorporate CAM-related discussions into survivorship care, and further studies are warranted to assess the impact of CAM modalities on quality of life and clinical outcomes.

## Introduction

The global aging trend, accelerating at an unprecedented pace, presents formidable challenges to healthcare systems worldwide. According to the World Health Organization, the elderly population is projected to rise dramatically to 2.1 billion by 2050 [1]. This demographic shift is particularly pronounced in countries like Japan and Korea, where the elderly are expected to comprise 38% and 46.4% of the population, respectively, by 2070, which will have a profound impact on their healthcare systems [2]. Such shift significantly strains healthcare resources and amplify the burden of diseases like cancer, which show increasing prevalent among the elderly [3]. Elderly cancer patients, due to their heightened vulnerability to treatment-related side effects, face compounded challenges [4]. In Europe, the widespread use of dietary supplements among over half of cancer patients necessitates proactive intervention by healthcare providers [5]. In Korea, lifestyle factors such as smoking and poor dietary habits place elderly men at greater risk, underscoring the urgency for targeted self-management investigations [6].

Elderly cancer patients have complex needs, often intertwined with mental health problems, as assesses by the Hospital Anxiety and Depression Scale (HADS) [17–20]. Beyond physical symptom management, complementary and alternative medicine (CAM) offers potential relief for emotional and depressive symptoms commonly experienced by cancer patients [21–24]. CAM modalities, including vitamins, ginseng, green tea, and yoga are widely embraced for their perceived efficacy in enhancing immune response, alleviating stress, and managing a variety of health conditions as cancer, cholesterol reduction, and digestive symptoms [28, 29]. Notably, ginseng and green tea have been used as therapeutic interventions for heart disease, anxiety, and depression [7]. Additionally, yoga is recognized for its ability to improve cellular function through by stress reduction, boosting immune function, and enhances physical attributes like flexibility, strength, and balance, thereby thus providing significant benefits to individuals undergoing cancer treatment [8].

Given these complexities, integrating CAM into supportive care for elderly cancer patients is imperative. Evidence suggests that those who engage in CAM practices, including spiritual practices such as personal prayer and meditation, report improved psychological well-being and quality of life [9]. Research indicates that personalized CAM interventions, aligned with patients’ cultural and individual preferences, led to better adherence to treatment plans and mitigate anxiety and depression [10]. This underscores the need for a holistic, patient-centered approach that respects the diverse practices and beliefs of elderly cancer patients.

Despite the potential benefits, supportive care incorporating CAM for elderly cancer patients remains scare. The integration of complementary medicine into conventional cancer care remains limited, complicating medical decision-making. Geographic and cultural contexts significantly influence CAM utilization patterns among cancer patients [11]. While certain CAM practices are prevalent in specific regions, such as mushroom usage in Japan and Chinese medicine in China, and diet and acupuncture are commonly embraced in Taiwan [12, 13], comprehensive studies focusing on CAM use among elderly cancer patients in major Asian nations remain limited. Therefore, the objectives of this study were (1) to investigate the patterns and characteristics of CAM use among elderly male cancer patients in Korea and (2) to identify factors associated with CAM utilization in this population.

## Materials and Methods

### 1. Setting and design

This study was conducted at the Veterans Hospital System (VHS) in Seoul, South Korea, targeting outpatients aged 65 years and older. A convenience sampling method was used, and cancer patients receiving care in six outpatient departments were surveyed: hematology-oncology, surgery, day wards, radiation oncology, gastroenterology, and urology. During the recruitment period, 600 eligible patients were identified, and 450 were invited to participate through both an invitation letter and a follow-up phone call. Of these, 420 agreed to participate (participation rate: 93.3%), while 30 declined. After excluding 12 incomplete questionnaires, data from 408 participants were included in the final analysis (response rate: 97.1%).

### 2. Study population and survey administration

Eligible participants were cancer patients aged 65 years or older who had been diagnosed within the past five years, were able to understand and respond to interview questions, and voluntarily agreed to participate. Patients with physical or cognitive impairments that hindered completion of the questionnaire were excluded. Using a convenience sampling strategy, participants were recruited consecutively in the outpatient setting until the target sample size was reached within the study timeframe. A total of 420 participants were enrolled, enabling the study to address the research questions effectively. While the sample may not be fully representative of the broader elderly cancer population, it was considered appropriate for hypothesis generation and exploratory analysis.

### 3. Dependent variables

The final questionnaire comprised four sections with a total of 34 items. Prior to assessing CAM use, the definition of complementary and alternative medicine (CAM) provided by the World Health Organization was presented to all participants to ensure clarity and consistency. Respondents were asked about their CAM utilization and the specific types of CAM used, which were categorized into five major groups. Additional questions explored reasons for CAM use, consultation with a physician regarding CAM, methods of accessing CAM, and associated out-of-pocket costs. For non-users, reasons for not using CAM were recorded. Finally, participants’ CAM-related practices were evaluated [14, 15].

### 4. Independent variables

The questionnaire assessed patients’ health status and disease characteristics, including relevant clinical features [23]. Depression and anxiety were measured using the Hospital Anxiety and Depression Scale (HADS), following the guidelines of the National Comprehensive Cancer Network (NCCN). Stress and pain were evaluated using the Korean version of the distress thermometer and problem list, developed in alignment with the NCCN Guidelines (version 2.2021). Additional items captured information on the cancer treatment process, including diagnosis, stage, and treatment modalities, as well as participants’ experiences with healthcare services.

### 5. Covariates

The first section of the questionnaire collected demographic and socioeconomic characteristics, including age, education level, marital status (married, divorced, widowed), and monthly household income [45, 46]. The data were collected from cancer patients attending six outpatient clinics between 25 August and 15 September 2021. Face-to-face interviews were conducted by trained interviewers, each lasting approximately 10–15 minutes. As a token of appreciation, participants who completed the survey received a $10 gift voucher.

### 6. Statistical analysis

All analyses were conducted using the Statistical Package for the Social Sciences (SPSS) version 26.0 (IBM Corp., Armonk, NY, USA). Descriptive statistics were used to summarize sociodemographic and clinical characteristics of the study population. Differences between CAM users and non-users were assessed using the chi-square test. Variables showing statistical significance in univariate analysis were further examined using logistic regression. Multiple logistic regression was performed to identify independent factors associated with CAM use. Statistical significance was set at p < 0.05.

## Results

### 1. Characteristics of the participants

The sociodemographic and clinical characteristics of the 408 participants are presented in Table 1. Nearly half of participants were 75 to 79 years of age (45.8%). Most participants had attained a high school level of education (39.9%) and were married or in a marital relationship (79.9%). A statistically significant association was identified between marital status and the utilization of complementary and alternative medicine (CAM), with CAM use being more common among those with a spouse (p = 0.002).

Prostate cancer was the most common diagnosis among participants (41.1%), followed by lung cancer (15.5%). Regarding treatment, 52.4% of participants had undergone surgical therapy and 46.3% had received chemotherapy. CAM utilization was significantly higher among patients diagnosed with prostate cancer (51.0%, p = 0.001) and among those who had undergone surgical treatment (56.7%, p = 0.043).

An analysis of psychological status demonstrated that CAM users reported significantly lower levels of anxiety (p = 0.016), but higher levels of depression (p = 0.014) compared to non-users. Although CAM use was more frequent among those with a distress score ≥ 4 (63.7%) compared with those scoring < 4 (36.3%) (36.3%), this difference was not statistically significant.

### 2. The types of CAM used and reasons

The distribution of CAM modalities among elderly cancer patients who reported CAM use is presented in Table 2. Among CAM users, fast walking is the most prevalent CAM intervention, reported by 49.4% of the patients. This was followed by dietary modifications (42.9%) and the use of nutritional supplements such as vitamins (35.1%) and omega-3 fatty acids (28.2%).

### 3. Reasons for CAM Use and Sources of Information

The reasons for CAM utilization among elderly cancer patients are summarized in Table 3. The most frequently reported motivation was to strengthen immunity (61.6%), followed by improving quality of life (30.6%). Other reported reasons included the belief that CAM is natural and without side effects (26.1%), prevention of cancer recurrence (15.1%), treatment of cancer itself (12.7%), and prolonging life (8.6%).

Sources of CAM information (n = 338) varied considerably. Family or relatives were the most common source (42.1%), followed by media such as television (news or advertisements) and newspapers (28.5%). Medical staff (17.8%), internet sources such as YouTube (16.5%), and neighbors or friends (16.1%) also contributed notably.

### 4. Factors influencing participants’ use of CAM

The results of the multivariate logistic regression analysis are presented in Table 4. Elderly cancer patients with a spouse were significantly more likely to use CAM than those without a spouse also positively associated with CAM utilization (OR = 2.493, *p* = 0.002). A diagnosis of prostate cancer (OR = 2.136, *p* = 0.001). Similarly, patients who had undergone surgical treatment were more likely to use CAM (OR = 1.615, 95% CI = 1.065–2.449, p = 0.024).

In contrast, perceived anxiety, depression, and distress levels were not significantly associated with CAM use in the adjusted model.

## Discussion

This study provides novel evidence on the prevalence and determinants of CAM use among elderly cancer patients in Korea, a population often underrepresented in supportive care research. The overall CAM utilization rate was 64%, considerably higher than previously reported rates, such as 27% among U.S. veterans with cancer and 51% in a recent global systematic review [16, 17]. Multivariate analysis identified marital status, surgical treatment, and prostate cancer diagnosis as significant predictors of CAM use. Notably, the high uptake of CAM among elderly Korean men contrasts with earlier findings that male patient generally less likely to engage in CAM [18]. This discrepancy may reflect sociocultural dynamics unique to Korea, including the influence of family caregiving patterns and strong cultural trust in traditional medicine. The importance of proactive clinician–patient dialogue regarding CAM, particularly with elderly male patients, and underscore the need to integrate culturally sensitive supportive strategies into cancer care [19].

Our study highlights important implications for supportive cancer care in elderly patients. Exercise and dietary interventions emerged as the most practiced CAM modalities, with fast walking reported most frequently. Lifestyle-based strategies such as exercise are not only feasible and safe but are also perceived positively by older patients, who report benefits including reduced stress, improved sleep, and enhanced quality of life [20]. Elderly cancer patients in particular perceive exercise positively, reporting improvements in physical health and emotional well-being, particularly in stress reduction and sleep quality [21]. These findings are consistent with prior evidence demonstrating improvements in physical function, disability reduction, chronic disease prevention, and psychological health [17]. In the Korean context, access to public exercise facilities and senior centers further facilitates participation, fostering social engagement and reducing isolation [22]. Collectively, these results underscore the need to integrate low-barrier, culturally appropriate lifestyle practices into survivorship care, reinforcing the holistic role of CAM in improving psychosocial well-being and clinical outcomes for elderly cancer patients.

Depression and anxiety symptoms are highly prevalent among elderly cancer patients and significantly affect treatment adherence, clinical outcomes, and overall quality of life [23]. In our study, CAM utilization was more common among patients with depressive symptoms than in those with higher anxiety, suggesting that depression may serve as a stronger motivator for seeking CAM adoption. Approximately two-thirds of participants reported borderline depressive symptoms, underscoring the substantial emotional burden in this population. These findings align with previous research suggesting that psychological vulnerability, particularly depression, often drives engagement with CAM as a form of self-empowerment and emotional regulation. Given that cancer-related fatigue (CRF) often exacerbates mood disturbances—particularly depression—non-pharmacological strategies like structured physical activity may serve as both preventive and therapeutic tools [24]. Collectively, these results highlight the importance of integrating CAM into holistic, patient-centered cancer care, with tailored exercise and dietary approaches playing a critical role in improving psychosocial well-being and supporting favorable clinical outcomes among elderly patients.

Participants in this study reported using CAM primarily to boost immunity and enhance quality of life, often perceiving this intervention as safer due to their natural origin. This perception aligns with previous research that elderly cancer patients frequently turn to CAM to manage symptoms, improve well-being, and assert greater control over treatment decisions [25, 26]. However, our findings also revealed several concerns regarding the potential risks associated with unsupervised CAM use in this population, including adverse interactions with conventional medications, increased toxicity, and diminished therapeutic efficacy of standard cancer treatments [27]. For instance, ginseng - a commonly used CAM product- has demonstrated cytotoxic effects on certain cancer cells, yet its unregulated use may lead to unpredictable outcomes [28]. Similarly, excessive consumption of dietary supplements such as vitamins can lead to complications including nephrolithiasis and hypercalcemia, thereby interfering with standard cancer therapies [29]. These risks underscore the limitations of CAM in elderly cancer care, where unregulated production standards, drug-herb interactions, and inconsistent evidence regarding efficacy remain critical concerns [30]. To mitigate these risks, healthcare providers should play a proactive role by offering balanced, evidence-based guidance on CAM use. Open, non-judgmental communication is crucial in supporting elderly patients in making informed and safe treatment decisions [25]. Furthermore, establishing trustworthy and accessible sources of CAM information is vital to ensure that elderly cancer patients can navigate integrative care safely and effectively.

The sources of CAM information among elderly cancer patients are diverse and shaped by interpersonal and media influences. Family members, spouses, and friends were the primary sources of CAM- related knowledge, aligning with prior studies highlighting the role of familial support in healthcare decisions. Traditional media, such as television programs and newspaper articles, continue to influence perceptions, while digital platforms, including YouTube, are emerging as modest but growing sources of information among older adults. Our findings highlight the significant role of family dynamics in facilitating CAM engagement, particularly in cases where a spouse is present, consistent with literature showing that families of cancer patients often express a heightened need for caregiving-related information and support.

At the same time, the proliferation of CAM-related information through non-clinical sources presents a critical challenge, as healthcare providers currently lack robust mechanisms to evaluate the safety, effectiveness, and compatibility of such information with standard oncological treatment regimens. Emerging research calls for healthcare professionals to play a pivotal role by establishing clinical guidelines and rationales for CAM use, thereby helping patients navigate the growing influx of informal health advice. Although cancer management policies are currently increasingly accessible online, they often fall short of addressing the nuanced needs of elderly populations. To bridge this gap, primary medical institutions should integrate exercise therapy and structured CAM education as default components of elderly cancer care protocols. Such institutional embedding, coupled with open clinician-patient dialogue, can enhance decision-making, mitigate treatment risks, and support the safe and informed incorporation of CAM into conventional cancer care.

This study has several limitations. Its cross-sectional design precludes causal inference, and the data were collected in 2021; patterns of CAM use may have changed since then due to evolving healthcare access, patient preferences, or the impact of the COVID-19 pandemic. Nevertheless, CAM utilization among elderly cancer patients remains highly relevant, and our findings provide timely evidence for supportive care planning. The single-institution, predominantly male veteran sample may limit generalizability, and reliance on self-reported questionnaires introduces recall bias. Moreover, details on dosage, duration, and frequency of CAM practices were not captured, and potential confounders such as socioeconomic status and comorbidities were not fully addressed. Future research should employ multicenter, longitudinal designs with updated datasets and incorporate clinical endpoints to clarify the impact of CAM on adherence, survivorship, and quality of life.

## Conclusion

This study emphasizes that elderly cancer patients in Korea widely use CAM for supportive care, with exercise and dietary interventions being the most common methods. Immune enhancement is a primary motivation for CAM use, often influenced by family support. Factors such as marital status, prostate cancer diagnosis, and surgical therapy are linked to CAM utilization. However, further research is needed to understand how these factors affect treatment outcomes. Ultimately, this will help develop guidelines to integrate CAM with conventional cancer care and improve supportive care for elderly patients.

## Figures and Tables

**Table 1:** Sociodemographic and Clinical Characteristics of Participants (n = 408)

Variables	Total N (%)	Users (N = 245)	Non-user (N = 163)	*P*-value
Age				
Mean ± SD	75.85 ± 5.87	75.96 ± 5.40	75.74 ± 6.34	
65–74	161 (39.4)	95 (38.8)	66 (40.4)	0.717
75–79	187 (45.8)	116 (47.3)	71 (43.6)	
≥ 80	60 (14.8)	34 (13.9)	26 (16.0)	
Educational level				
Below middle school	155 (38.0)	92 (37.6)	63 (38.7)	0.752
High school	163 (39.9)	99 (40.4)	67 (41.1)	
College level and above	87 (22.1)	54 (22.0)	33 (20.2)	
Marital status				
Spouse	326 (79.9)	184 (75.1)	142 (87.1)	**0.002** **
No spouse ^a^	82 (20.1)	61 (24.9)	21 (12.9)	
Monthly Income				
≤ 1,000,000 (KRW)	126 (30.8)	69 (28.1)	57 (35.0)	0.752
1,000,000–3,000,000 (KRW)	225 (55.1)	143 (58.3)	82 (50.3)	
≥ 3,000,000 (KRW)	57 (14.1)	33 (13.6)	24 (14.7)	
Perceived health status				
Good	203 (49.7)	121 (49.4)	82 (50.3)	0.856
Bad	205 (50.3)	124 (50.6)	81 (49.7)	
Type of cancer^b^ (N = 445)				
Prostate cancer	183 (41.1)	125 (51.0)	58 (35.6)	**0.001** **
Lung cancer	69 (15.5)	35 (14.3)	34 (20.9)	0.056
Rectal, colon cancer	40 (8.9)	22 (9.0)	18 (11.0)	0.301
Stomach cancer	33 (7.4)	18 (7.3)	15 (9.2)	0.310
Liver cancer	24 (5.3)	15 (6.1)	9 (5.5)	0.490
Thyroid cancer	3 (1.0)	1 (0.4)	2 (1.2)	0.351
Others	93 (20.8)	53 (21.6)	40 (24.5)	0.285
Type of treatment ^c^				
Surgery	214 (52.5)	139 (56.7)	75 (46.0)	**0.043** *
Chemotherapy	189 (46.3)	111 (45.3)	78 (47.9)	0.685
Radiotherapy	143 (35.0)	81 (33.1)	62 (38.0)	0.341
Hormone therapy	48 (11.8)	32 (13.1)	16 (9.8)	0.350
Others	44 (10.8)	30 (12.2)	14 (8.6)	0.259
Perceived anxiety level ^d^				
0–7 points	267 (65.4)	169 (68.9)	98 (60.1)	**0.016** *
8–21 points	141 (34.6)	76 (31.1)	65 (39.9)	
Perceived depression level ^d^				
0–7 points	105 (25.7)	73 (29.8)	32 (19.6)	**0.014** *
8–21 points	303 (74.3)	172 (70.2)	131 (80.4)	
Perceived distress level				
< 4 points	159 (39.0)	89 (36.3)	70 (42.9)	0.108
≥ 4 points	249 (61.0)	156 (63.7)	93 (57.1)	

* P < 0.05

** P < 0.001

a Unmarried variable included single/divorced/separated/widowed.

b Type of cancer: multiple responses allowed.

c Type of treatment: multiple responses allowed.

d Normal: 0–7 points, Borderline: 8–10 points, Caseness: 11–21 points

**Table 2:** Types of Complementary and Alternative Medicine (CAM) Used by Cancer Patients (n = 245)

Used CAM modalities ^a^	N (%)
Natural product	
Dietary (black beans, Mixed grains)	105 (42.9)
Vitamins	86 (35.1)
Omega3	69 (28.2)
Ginseng, Red ginseng	55 (22.4)
Mushrooms (Chaga, Phellinus linteus)	31 (12.7)
Baked garlic	13 (5.3)
Green tea or herbal	12 (4.9)
Others	63 (25.7)
Mind and body practices	
Fast walking	121 (49.4)
Massage	6 (2.4)
Hypogastric breathing	2 (0.8)
Manual therapy	1 (0.4)
Pray, Meditation	10 (4.1)
Psychotherapy	5 (2.0)
Other complementary health approaches	
Hand acupuncture	7 (2.9)
Acupressure therapy	3 (1.2)
Fenbendazole	1 (0.4)
Canine meat soup	1 (0.4)

a Multiple responses allowed.

* Columns do not sum to 100% due to multiple responses.

**Table 3:** Reasons for CAM Use and Sources of CAM Information Among Cancer Patients.

Category*	N (%)
Reasons for using CAM (n = 393) ^a^	
Strengthen immunity	151 (61.6)
Improve the quality of life	75 (30.6)
Belief that CAM is natural/ no side effect	64 (26.1)
Prevent recurrent of cancer	37 (15.1)
To treat cancer	31 (12.7)
To live longer	21 (8.6)
Others	14 (5.6)
Sources of CAM information (n = 338) ^a^	
Family or relatives	102 (42.1)
TV (news or advertisement) / Newspaper	69 (28.5)
Medical staffs (doctors, nurses)	43 (17.8)
Internet (YouTube)	40 (16.5)
Neighbors or Friends	39 (16.1)
Other patients	11 (4.5)
Pharmacist	5 (2.1)
Literature	5 (2.1)
Traditional Korean Medicine Hospitals	1 (0.4)
Others	23 (9.5)

a Multiple responses allowed; therefore, column percentages do not total 100%.

**Table 4:** Multivariate Logistic Regression Analysis of CAM Use by Demographic and Clinical Factors

Category	OR	95% CI	*P*-value
Marital status			
No Spouse	1	Ref.	
Spouse	2.493	1.417–4.384	**0.002** *
Type of cancer			
Non-prostate cancer	1	Ref.	
Prostate cancer	2.136	1.394–3.273	**0.001** *
Surgery treatment			
No	1	Ref.	
Yes	1.615	1.065–2.449	**0.024** *
Perceived Anxiety			
0–7 points	1	Ref.	
Above 8 points	0.839	0.530–1.328	0.454
Perceived Depression			
0–7 points	1	Ref.	
Above 8 points	0.654	0.389–1.100	0.110
Perceived distress level			
≥ 4 points	1	Ref.	
< 4 points	1.435	0.939–2.914	0.095

OR = Odds ratio; CI = Confidence interval; Ref. = reference category.

* Statistically significant at P < 0.05.

## Data Availability

The data supporting this study’s findings are available from the corresponding author. Access to the data may be subject to privacy and ethical considerations.

## References

[1] World Health Organization. Ageing 2024 [cited 2024 (n.d)]; Available from: https://www.who.int/health-topics/ageing#tab=tab_1.

[2] KOSIS. Korea in the world 2024 [cited 2024 June 08]]; Available from: KOSIS KOrean Statistical Information Service.

[3] Torres A.C., García-Valdés P., and Henriquez-Santos G., Geriatric emergencies in older adults with cancer: why older patients need a special approach. Gaceta mexicana de oncología, 2023. 22(1): p. 41–51.

[4] Mudaranthakam D.P., , Financial burden among cancer patients: A national-level perspective. Cancer Medicine, 2023. 12(4): p. 4638–4646. 35852258 10.1002/cam4.5049 PMC9972087

[5] Kristoffersen A.E., , Exploring dietary changes and supplement use among cancer patients in Norway: prevalence, motivations, disclosure, information, and perceived risks and benefits: a cross sectional study. BMC Nutrition, 2024. 10(1). 10.1186/s40795-024-00872-8 PMC11055316 38671478

[6] Seo H.-J., , Associations of financial toxicity with employment concerns and cancer-related distress: a cross-sectional survey among korean working-age cancer survivors. Cancer Research and Treatment: Official Journal of Korean Cancer Association, 2024. 57(3): p. 659. 10.4143/crt.2024.090 PMC12263219 39638252

[7] Vet O., , Cancer Therapy Interactions with Foods and Dietary Supplements. PDQ Cancer Information Summaries; National Cancer Institute: Bethesda, MD, USA, 2022.

[8] Agarwal R.P. and Maroko-Afek A., Yoga into cancer care: a review of the evidence-based research. International journal of yoga, 2018. 11(1): p. 3. 29343927 10.4103/ijoy.IJOY_42_17 PMC5769195

[9] Movafagh A., , Spiritual Therapy in Coping with Cancer as a Complementary Medical Preventive Practice. Journal of Cancer Prevention, 2017. 22(2): p. 82–88. 28698861 10.15430/JCP.2017.22.2.82 PMC5503219

[10] McDermott C.L., , Complementary and alternative medicine use among newly diagnosed prostate cancer patients. Supportive Care in Cancer, 2012. 20: p. 65–73. 21120540 10.1007/s00520-010-1055-y

[11] Abuelgasim K.A., , The use of complementary and alternative medicine by patients with cancer: a cross-sectional survey in Saudi Arabia. BMC complementary and alternative medicine, 2018. 18(1): p. 1–8. https://bmccomplementmedtherapies.biomedcentral.com/counter/pdf/10.1186/s12906-018-2150-8.pdf 29530034 10.1186/s12906-018-2150-8 PMC5848536

[12] Lin Y.-H., Chen K.-K., and Chiu J.-H., Prevalence, patterns, and costs of Chinese medicine use among prostate cancer patients: a population-based study in Taiwan. Integrative cancer therapies, 2010. 9(1): p. 16–23. 20308084 10.1177/1534735409359073

[13] Chin C.-Y., , Complementary and alternative medicine use in breast cancer patients at a medical center in Taiwan: a cross-sectional study. Integrative cancer therapies, 2020. 19: p. 1534735420983910. 33372560 10.1177/1534735420983910 PMC7797811

[14] Hwang J.H., , Complementary and alternative medicine use among outpatients during the 2015 MERS outbreak in South Korea: a cross-sectional study. BMC complementary medicine and therapies, 2020. 20(1): p. 1–10. 32404092 10.1186/s12906-020-02945-0 PMC7220580

[15] Oyunchimeg B., , Complementary and alternative medicine use among patients with cancer in Mongolia: a National hospital survey. BMC complementary and alternative medicine, 2017. 17(1): p. 1–8. https://bmccomplementmedtherapies.biomedcentral.com/counter/pdf/10.1186/s12906-017-1576-8.pdf 28103860 10.1186/s12906-017-1576-8 PMC5244576

[16] Dunya Mahmoud E.J.E.B., (Loes) Visser L.E., (Anthonius) de Boer A., and c.p.a.e. B, The Prevalence of Clinically Relevant Herb-Drug Interactions between Herbal Products and Anti-Cancer Therapy in Older Adults with Cancer. 2021. 10.1016/j.rcsop.2025.100585 PMC11982495 40212889

[17] Kaur H., , Dietary Supplement Use among Older Cancer Survivors: Socio-Demographic Associations, Supplement Types, Reasons for Use, and Cost. Nutrients, 2022. 14(16): p. 3402. 10.3390/nu14163402 36014907 PMC9414522

[18] Wode K., , Cancer patients’ use of complementary and alternative medicine in Sweden: a cross-sectional study. BMC Complementary and Alternative Medicine, 2019. 19(1). https://bmccomplementmedtherapies.biomedcentral.com/counter/pdf/10.1186/s12906-019-2452-5.pdf 10.1186/s12906-019-2452-5 PMC6417272 30866916

[19] Filbet M., , The use of complementary medicine in palliative care in France: an observational cross-sectional study. Supportive Care in Cancer, 2020. 28(9): p. 4405–4412. 31919664 10.1007/s00520-020-05296-1

[20] Miura S., , A randomized phase II study of nutritional and exercise treatment for elderly patients with advanced non-small cell lung or pancreatic cancer: the NEXTAC-TWO study protocol. BMC Cancer, 2019. 19(1). 10.1186/s12885-019-5762-6 PMC6544995 31151425

[21] McCall M., Ward A., and Heneghan C., Yoga in adult cancer: a pilot survey of attitudes and beliefs among oncologists. Current Oncology, 2015. 22(1): p. 13–19. 25684984 10.3747/co.22.2129 PMC4324339

[22] Lee E.J. and Park S.J., Immersive experience model of the elderly welfare centers supporting successful aging. Frontiers in psychology, 2020. 11: p. 8. https://pmc.ncbi.nlm.nih.gov/articles/PMC7026025/32116886 10.3389/fpsyg.2020.00008 PMC7026025

[23] Trevino K.M., Saracino R.M., and Roth A.J., Symptomatology, assessment, and treatment of anxiety in older adults with cancer. Journal of Geriatric Oncology, 2021. 12(2): p. 316–319. 32565145 10.1016/j.jgo.2020.06.011 PMC7303031

[24] Kraft K., CAM for depression, anxiety, grief, and other symptoms in palliative care. Progress in Palliative Care, 2012. 20(5): p. 272–277.

[25] Källman M., , Use of CAM among cancer patients. BMC Complementary Medicine and Therapies, 2023. 23(1). 10.1186/s12906-023-03876-2 PMC9933304 36797715

[26] Wode K., , Cancer patients’ use of complementary and alternative medicine in Sweden: a cross-sectional study. BMC complementary and alternative medicine, 2019. 19(1): p. 1–11. 30866916 10.1186/s12906-019-2452-5 PMC6417272

[27] Hersh L.R., Beldowski K., and Hajjar E.R., Polypharmacy in the Geriatric Oncology Population. Current Oncology Reports, 2017. 19(11). 10.1007/s11912-017-0632-3 28942563

[28] Majeed F., , Ginseng phytochemicals as therapeutics in oncology: Recent perspectives. Biomedicine & Pharmacotherapy, 2018. 100: p. 52–63. 29421582 10.1016/j.biopha.2018.01.155

[29] Mangione C.M., , Vitamin, Mineral, and Multivitamin Supplementation to Prevent Cardiovascular Disease and Cancer. JAMA, 2022. 327(23): p. 2326. https://www.aafp.org/pubs/afp/issues/2022/1100/ppip-supplementation-cardiovascular-disease-cancer.pdf 35727271 10.1001/jama.2022.8970

[30] River J., , Convergent priorities and tensions: a qualitative study of the integration of complementary and alternative therapies with conventional cancer treatment. Supportive Care in Cancer, 2018. 26(6): p. 1791–1797. https://link.springer.com/content/pdf/10.1007/s00520-017-4021-0.pdf 29249059 10.1007/s00520-017-4021-0

